# Multivariate search for differentially expressed gene combinations

**DOI:** 10.1186/1471-2105-5-164

**Published:** 2004-10-26

**Authors:** Yuanhui Xiao, Robert Frisina, Alexander Gordon, Lev Klebanov, Andrei Yakovlev

**Affiliations:** 1 Department of Biostatistics and Computational Biology, University of Rochester, 601 Elmwood Avenue, Rochester, New York 14642, USA; 2 Departments of Otolaryngology, Neurobiology and Anatomy, and Biomedical Engineering, University of Rochester, 601 Elmwood Avenue, Rochester, New York 14642, USA; 3 Department of Probability and Statistics, Charls University, Sokolovska 83, Praha-8, CZ-18675, Czech Republic

## Abstract

**Background:**

To identify differentially expressed genes, it is standard practice to test a two-sample hypothesis for each gene with a proper adjustment for multiple testing. Such tests are essentially univariate and disregard the multidimensional structure of microarray data. A more general two-sample hypothesis is formulated in terms of the joint distribution of any sub-vector of expression signals.

**Results:**

By building on an earlier proposed multivariate test statistic, we propose a new algorithm for identifying differentially expressed gene combinations. The algorithm includes an improved random search procedure designed to generate candidate gene combinations of a given size. Cross-validation is used to provide replication stability of the search procedure. A permutation two-sample test is used for significance testing. We design a multiple testing procedure to control the family-wise error rate (FWER) when selecting significant combinations of genes that result from a successive selection procedure. A target set of genes is composed of all significant combinations selected via random search.

**Conclusions:**

A new algorithm has been developed to identify differentially expressed gene combinations. The performance of the proposed search-and-testing procedure has been evaluated by computer simulations and analysis of replicated Affymetrix gene array data on age-related changes in gene expression in the inner ear of CBA mice.

## Background

The set of microarray expression data on *p *distinct genes is represented by a random vector **X **= *X*_1_,..., *X*_*p *_with stochastically dependent components. The dimension of **X **is typically very high relative to the number of observations (replicates of experiment). The standard practice is to test the hypothesis of no differential expression for each gene. Formulated in terms of the marginal distributions of all components of **X**, this hypothesis means that the expression levels of a particular gene are identically distributed under two (or more) experimental conditions. It is commonly believed that the only challenging problem here is that of multiple statistical tests, because the corresponding test statistics computed for different genes are stochastically dependent. This problem is discussed in [2] in the context of microarray data analysis. Resampling techniques [3,4] provide a universal approach to the problem of multiple dependent tests inherent in the most typical study designs. However, there is another aspect of the standard approach that warrants special attention. Any test constructed solely in terms of marginal distributions of gene expression levels disregards the multidimensional (dependence) information hidden in gene interactions, which is its most obvious deficiency.

In a recent paper, Szabo et al. [5] proposed to build a target set of interesting genes from non-overlapping subsets of genes of a given size (≥1) that have been declared differentially expressed in accordance with a pertinent statistical test. The size of each sought-for subset is naturally constrained by the available sample size. This approach strives to preserve the dependence structure at least within each of such building blocks, which is already a major step toward a more general methodology of microarray gene expression data analysis.

No matter what specific statistical techniques are chosen to approach the problem of identifying differentially expressed gene combinations rather than individual genes, the hypothesis that the expression levels of a given set of genes are identically distributed across the conditions under study is the most meaningful hypothesis to be tested. However, this hypothesis is now formulated in terms of the joint distribution of expression levels. The issue of multiple testing is dramatically magnified with multivariate methodology, because the total number of tests to be carried out at all steps of multivariate selection may be many orders of magnitude larger than with univariate methods. A constructive idea is to design a random search procedure for identifying differentially expressed sets of genes followed by testing significance of a final set. Szabo et al. [5,6] proposed a search procedure based on maximization of a new distance between multivariate distributions of gene expression signals. They used permutation techniques for hypotheses testing. To adjust for multiple testing, the null-distribution was estimated from the test statistics generated by each optimal (in terms of the adopted distance) set of genes found in each permutation sample. The authors provided an illustrative example of clear advantages of multivariate methodology over univariate approaches. In the present paper, we improve the cross-validation and multiple testing components of the earlier proposed algorithm. This new combination of the search-and-testing procedures furnishes a sound statistical methodology for multivariate analysis of microarray data.

## Results

### Mathematical framework: measure of differential expression

To compare gene expression signals in two different experimental conditions (states) one needs a pertinent distance between two random vectors. Such a distance is expected to satisfy the following requirements: (1) it should have a clear probabilistic meaning; (2) it should accommodate both continuous and categorical data; (3) its estimate should be stable to random fluctuations and numerical errors; (4) its computation should not be too time consuming. A distance that meets all the above requirements was proposed in [6].

Let **X **= *X*_1_,..., *X*_*d *_and **Y **= *Y*_1_,..., *Y*_*d*_, *d *≤ *p*, be two random sub-vectors with probability measures *μ *and *ν*, respectively, defined on the Euclidean space **R**^*d*^. Let *K*(**x**, **y**) be a strictly negative definite kernel, that is  for any **x**_1_,..., **x**_*s *_from **R**^*d *^and any real numbers *h*_1_,..., *h*_*s*_, , with equality if and only if all *h*_*i *_= 0. Introduce the following expression



The quantity *N*(*μ*, *ν*) can be shown [7] to be a metric in the space of all probability measures **R**^*d*^, so that the null hypothesis in two-sample comparisons can be formulated as *H*_0 _: *N*(*μ*, *ν*) = 0. A normalized version of *N *can be derived as , where



If *K*(**x**, **y**) = Ψ(**x **- **y**) and Ψ(·) is homogeneous of any order, then *N*_norm _is both location and scale invariant.

Consider two independent samples, consisting of *n*_1_and *n*_2 _observations respectively, represented by the *d*-dimensional vectors  and , and introduce an empirical counterpart (nonparametric estimate) of *N*(*μ*, *ν*) as follows



A very important advantage of the empirical counterpart  of the distance *N *is that it does not involve numerically unstable high-dimensional components (such as covariance matrix or its inverse), thus it is expected to be numerically stable even for small sample sizes. This was corroborated by a computer simulation study [5], in which this distance demonstrated a much higher stability than the Mahalanobis distance and the nearest neighbor classifier.

Another distinct advantage of the approach based on  is a wide selection of negative definite kernels that are sensitive to various departures from the hypothesis: *μ *= *ν*. Let **x **and **y **denote observations in two samples on a particular set  of variables. We consider either of these observations to be points in Euclidean space **R**^*d*^. One natural choice is the Euclidean distance between points representing experimental measurements:



When this kernel is applied to logarithms of gene expression signals the corresponding distance  is scale invariant. Another possible choice is a bounded kernel exemplified by



Yet another kernel based on the correlation coefficient tends to pick up sets of genes with separated means and differences in correlation in the two samples under comparison [6]. One can also use a convex combination of the above mentioned kernels with the weights chosen in such a way as to make the distance more sensitive to particular types of the alternative hypothesis.

### The search-and-testing algorithm

Once a multivariate distance between expression signals has been selected, it can be employed in a search for differentially expressed genes with the target subset of genes being defined as a subset for which the distance between the two groups under comparison attains its maximum. Unlike univariate testing, an exhaustive multivariate search is computationally prohibitive because the number of possible subsets increases as the *d*-th power of the total number of genes. The issue of computational complexity can be resolved by applying random search methodology. Random search can be designed in a number of various ways. One simple algorithm was described in [6,8]. We used this algorithm, hereafter designated as Simple Random Search (SRS), with multiple random starts and long sequences of search steps in the application reported in the present paper. We also compared its performance with that of simulated annealing [1].

To reduce the selection bias associated with choosing a small number of variables from a large set [9], Szabo et al. [5,6] suggested to use cross-validation techniques with the search for a target subset of genes running in each cross-validation cycle. The basic structure of our cross-validation algorithm is as follows:

#### Algorithm A1: Cross-validated search for differentially expressed genes

1. Randomly draw (without replacement) *u*_1 _samples from one group of arrays and *u*_2 _samples from the other group.

2. Leave out the selected arrays and find the optimal (in accordance with the chosen criterion) subset of genes using only the data from the remaining arrays.

3. Repeat steps 1 and 2 in succession *v *times to obtain *v *"optimal" sets of genes.

The main problem here is that the algorithm results in many overlapping sub-optimal sets, and one needs to somehow combine them to report a single final set. Szabo et al. resorted to a somewhat unnatural way of forming a final set by selecting single genes with the highest frequencies of occurrence in sub-optimal sets. In our new algorithm, this is accomplished through designing a second-stage cross-validated search limited to the union of the previously selected sets. In the second-stage search procedure, cross-validation is carried out at each step of random search with the distance  averaged over all cross-validation samples. With this approach, the correlation structure is better preserved when combining the results of cross-validation. The foregoing description of the second-stage search may be summarized in the following algorithm:

#### Algorithm A2: The second-stage cross-validated random search

1. Form the union of all sets resulted from Algorithm A1 to represent an initial target set. Drop the data on all other genes from the data set.

2. Initiate a random search algorithm.

3. At each step of the search algorithm, randomly draw (without replacement) *l*_1 _samples from one group of arrays and *l*_2 _samples from the other group. Leave out the selected arrays and compute the *N*-statistic using only the data from the remaining arrays. Perform this computation *r *times.

4. Compute the average (arithmetic mean) of the *N*-statistics resulted from step 3. Denote this average by .

5. Move to the next step of random search using the statistic  as a pertinent objective function to be maximized.

In the application discussed in the present paper, we used Algorithm A2 with 200 cross-validated samples in the second stage of the search algorithm. The two-stage search algorithm runs with multiple random starts and returns the most differentially expressed (in terms of the distance ) gene combination of a given size.

Once an optimal set has been found, all genes pertaining to this set are discarded and a search for the next set of differentially expressed genes is initiated. Szabo et al. [5] proposed a stopping rule based on a permutation significance test. In the improved version of our algorithm, instead of testing significance at each step of the successive selection of subsets of genes, the selection procedure runs (without testing) for a preset number of steps, thereby forming a reasonably long sequence of non-overlapping "maximal" subsets. The same cross-validated random search procedure is applied to each permutation sample, generated to model the complete null hypothesis for disjoint subsets of genes, and finally the step-down multiple testing resampling algorithm by Westfall and Young [4] is applied to the subsets thus selected. If all the null hypotheses happen to be rejected, the selection procedure goes on eliminating subsets of genes resulting from the search algorithm, otherwise the procedure stops. The heuristic procedure thus designed mimics its univariate multiple testing (marginal hypotheses testing) counterpart with known properties [4], thereby ensuring an approximate control of the family-wise error rate (FWER).

Suppose that all tests are two-tailed and utilize the same test statistic , then the following resampling algorithm can be proposed:

#### Algorithm A3: Successive selection of differentially expressed gene combinations

1. Form *m *permutation samples of sizes *n*_1_and *n*_2_, respectively, from *n*_1_+ *n*_2 _replicated observations (arrays). For each of the *m *permutation samples, run (without testing) the successive selection algorithm to find a preset number *I *of disjoint sets. At each step of successive selection, an optimal *k*-element set is identified by the two-stage cross-validated search algorithm and the corresponding *m *sequences of -values are stored.

2. Returning to the original two-sample setting, find a sequence of *I *optimal sets of the same size *k *and compute the respective test statistics  for the selected sets.

3. Apply the step-down multiple testing resampling algorithm by Westfall and Young [4] to the *N*-statistics resulting from Steps 1 and 2. If the number of rejected hypotheses is less than *I *then stop and declare all the rejected sets of genes differentially expressed, otherwise return to Step 1 and continue successively selecting sets of genes. A faster version of Step 3 uses the single-step resampling adjustment [4].

The above algorithm can be reformulated in terms of *p*-values. The algorithm is computationally more expensive than its prototype presented in [5]. We used a SunFire V480 station to implement the algorithm. This "brute force" approach is needed to extract more information from multivariate gene expression profiles.

With the above approach, no distributional assumptions are needed although the test statistic  is not distribution free. For this statistic, however, it can be proven that permutations produce samples from a distribution that is, in some sense, the *least favorable *for rejecting an underlying composite null hypothesis. In other words, permutations provide an optimal choice of a null distribution. More precisely, this theoretical result is valid for the resampling (with replacement) analog of permutations, but regular (without replacement) permutations may be a good approximation to this resampling procedure if both samples under comparison are not too small. This concept and its mathematical framework is discussed at length in our previous report [10].

For efficient nonparametric estimation of adjusted *p*-values associated with sets of genes resulting from random search, it is also desirable that the test statistic be scale invariant for any sample size. A statistic that meets this requirement is an empirical counterpart of the normalized distance *N*_norm _with a properly chosen kernel function, see formula (2) and the succeeding explanation. Yet another possibility is to use the kernel *K*_1 _with log-intensities of gene expressions. We employed the latter pivoting structure of the *N*-statistic in the analysis of simulated and biological data presented in the subsequent sections.

### Simulation studies

We first tested our methodology by computer simulations. To this end, we designed a simulation study as follows.

Two sets of data on 1,000 genes were simulated. For convenience we will label them as "control" and "treatment" samples, respectively. The size of each sample was equal to 10. In the treatment group, the first 12 genes were set to be differentially expressed. To simulate these genes, logarithms of gene expression signals were generated from a multivariate normal distribution with an exchangeable correlation structure. The algorithm designed to simulate such data is presented in the Appendix. The correlation coefficient for all pairs of gene log-intensities was set equal to 0.6, while the standard deviation was chosen to be either *σ *= 0.5 or *σ *= 1 for all individual genes. The mean log-expression values *τ *for the genes assigned to the target set of genes were specified as follows: *τ *= 5 for the first 4 genes (Subset 1), *τ *= 4 for the second group of 4 genes (Subset 2), *τ *= 3 for the third group of 4 genes (Subset 3). The remainder of the genes (not differentially expressed) were simulated as log-normally distributed random variables with *τ *= 1 and the same standard deviation (either *σ *= 0.5 or *σ *= 1) and correlation coefficient. The 1,000 genes in the control group were simulated just like those that were not differentially expressed in the treatment group.

Our search-and-testing procedure was applied to the data sets thus generated in order to see whether (and how frequently) it can find all subsets, as well as all individual genes, included in the target set of differentially expressed genes. In each experiment, the SRS algorithm was run with multiple random starts. At each step of the successive selection of genes, the algorithm sought for a subset of 4 genes. The parameter *I *in Algorithm A3 was set equal to 5. Since the sole purpose of our simulations was to check how well a given algorithm finds a maximum of the *N*-statistic over gene sets, no recourse to cross-validation was made in this study. The number of permutations was set at 200. Because such simulations are very time consuming the experiment was repeated only 100 times. Two samples (control and treatment) were generated in each of the 100 experiments.

First we tested the SRS algorithm with 8 random starts and 2,500 search steps. When *σ *= 0.5 for the treatment group the algorithm was able to correctly recover Subset 1 in 82%, Subset 2 in 72%, and Subset 3 in 76% of simulation runs. The proportion of cases where all 12 genes were correctly recovered (irrespective of the order they entered the selected subsets) was 61%. The false discovery rate, defined as the mean proportion of falsely discovered genes among the true differentially expressed genes, was equal to 0.02.

When *σ *= 1 the SRS algorithm recovers Subset 1 in 76%, Subset 2 in 56%, and Subset 3 in 39% of the simulation runs. The proportion of cases where all 12 genes were correctly recovered was 53%. The false discovery rate was equal to 0.04.

As one would expect, the SRS algorithm performed better with 16 random starts and 3,600 search steps. For *σ *= 0.5, the rate of correct discovery becomes 100% for all three sets. For *σ *= 1 the algorithm correctly recovers Subset 1 in 81%, Subset 2 in 65%, and Subset 3 in 48% of simulation runs. The proportion of cases where all 12 genes are correctly recovered is 62%. However, the false discovery rate remains essentially the same as when running the SRS algorithm with 8 starts and 2,500 search steps. The results on individual simulated genes are presented in Table 1.

By way of comparison, we ran the Westfall and Young algorithm with a univariate counterpart of the test statistic *N *at the same level of FWER. While the results for *σ *= 0.5 were identical (100% correct recovery), the univariate method recovered less genes (45%) in the target set when we set *σ *= 1. In the latter case, the univariate algorithm had a uniformly lower correct discovery rate for genes #9 through #12 (69%, 71%, 70%, 71%, respectively) in comparison to the multivariate method (Table 1). One should not expect much discrepancy between the univariate and multivariate methods in these simulations because the alternative hypotheses were modeled in a univariate way.

In another experiment we studied the simulated annealing optimization (SAO) with one random start and the same parameters of the simulation model. Although computationally expensive, the SAO algorithm is easier to handle when tuning its parameters in simulation experiments. Proceeding from the less favorable case of *σ *= 1, we determined parameters of the SAO algorithm that provide correct selection of all three sets of differentially expressed genes in all simulation runs.

Another way of testing the two algorithms is to apply them in a situation where the true global maximum of the *N*-distance is known. We randomly selected 2000 genes from the data set discussed in the next section. All possible pairs were formed from the 2000 genes and the corresponding *N*-statistic between the two samples (young versus old mice) was computed for each pair. The data were normalized before the analysis (see Section "Results and Discussion"). Having determined a maximum value of the *N*-statistic over all pairs, we ran the SRS and SAO algorithms (with parameters suggested by our simulation experiments) to see whether they could find the actual maximum. Both algorithms hit the target.

## Results

The biological purpose of our experimental study was to better understand age-related changes in gene expression that occur in mouse inner ear (including the organ of Corti and stria vascularis). Since we do not expect numerous genes to be involved in the process of aging of the auditory system, this experimental system seems to be especially promising for the use of multivariate methods.

Hearing loss or deafness affects about 10% of the U.S. population, or about 30 million people, most of them over age 60. Presbycusis – age-related hearing loss – is a primary sensory problem in the elderly population, the number one communicative disorder, and one of the top three chronic medical conditions affecting the aged. It is often described as difficulty in understanding speech, especially in conditions of high ambient background noise. Most elderly persons have a reduction in hearing acuity. For example, cross-sectional and longitudinal studies have consistently demonstrated gradually decreasing pure tone thresholds by cohort groups of elderly [13,14]. The composite audiometric pattern is one of better hearing for low- and mid-speech frequencies than higher speech frequencies. The consequence of this pattern is difficulty in hearing and understanding, not only conversational speech, but in particular, speech that is softly spoken. In fact, a similar gradual reduction in speech recognition for words and phonemes in quiet has been shown to accompany the pure tone threshold decrease in cohort groups of the elderly [14-16].

Much progress had been made in the field of auditory aging research regarding sensitivity deficits and metabolic problems of the cochlea. As humans and animals age, they lose sensory hair cells, 8th cranial nerve (i.e., vestibulocochlear) fibers, and develop stria vascularis/potassium recycling metabolic problems that degrade audibility and spectral tuning [17-21].

In addition, the differing roles of the ear and brain in presbycusis, and aging deficits in speech understanding in background noise, and their respective neural bases are beginning to be understood. Age effects in these areas are distinguishable and age-related problems in the brain can be influenced by the peripheral etiologies of presbycusis [22-24]. Considering studies completed to date, presbycusis in humans, and corresponding age-related hearing loss in animal models such as the CBA mouse, have two major facets: 1) A peripheral hearing loss of cochlear origin, starting with sensitivity losses in the high pitches (high frequencies), involving loss of sensory hair cells, spiral ganglion neurons (8th nerve fibers) and metabolic malfunctions of the highly vascularized stria vascularis organ system that produces the potassium rich endolymph of the inner ear [25,26]; and 2) An inability to comprehend speech in background noise, that results from deficits in the inner ear and the central auditory nervous system [23,24]. For the animal model studies of presbycusis, the CBA mouse strain has been quite useful to date.

The goal of the present study is to explore the underlying cochlear gene expression changes that may predispose or cause presbycusis. Common neurodegenerative diseases such as presbycusis are likely to be caused by several fundamental problems that interact with each other and with environmental factors, including genetic pre-dispositions to environmental insults, noise and ototoxic medications [27]. Although over a hundred genes have been identified that cause congenital deafness (e.g. [28-30]), no candidate genes have yet been identified that are involved in human presbycusis. The present report attempts to gain some initial insights into gene expression changes related to inner ear problems that may predispose or cause age-related neurosensory disorders, such as age-related hearing loss – presbycusis, utilizing the CBA mouse strain.

The two groups of arrays under comparison included 9 and 12 arrays, respectively (see the next section). The data were normalized using the quantile normalization method [11,12] carried out at the probe feature level. Compared to our simulations, the number of permutations was increased to 400. Each search cycle in the SRS algorithm proceeded in 45,000 steps with 100 random starts. The algorithm was tuned to search for a set of 5 genes at each step of the successive selection procedure. We also changed parameters that control the efficiency of the SAO algorithm to account for an increased dimensionality of the problem. The latter algorithm also sought for sets consisting of 5 genes. We used the following parameter values in the combined two-stage cross-validated search algorithm: *I *= 5, *u*_1 _= 4 (out of 9 arrays), *u*_2 _= 6 (out of 12 arrays), *v *= 10, *l*_1 _= 4 (out of 9 arrays), *l*_2 _= 6 (out of 12 arrays), *r *= 200.

Although the lists of genes produced by both algorithms are quite similar, there are still some discrepancies between them which may be attributed to the choice of parameters for each method. Since the SAO algorithm is less sensitive to the choice of the initial gene combination, we present only the results obtained with this algorithm. In the "young" versus "old" comparison, the procedure selected two sets of 5 genes with an adjusted *p*-value of less than 0.05. For comparison, we applied the Wesfall and Young step-down multiple testing procedure with a univariate counterpart of  as the test statistic. This method selects only 6 genes at the same FWER; all of them appear among those genes that have been selected by the multivariate search-and-testing procedure. The final list of 10 genes was evaluated further for consistency with the existing biological knowledge.

## Discussion

Of the 10 identified genes (from 2 sets) exhibiting major expression changes with age, there are 6 differentially expressed genes having to do with immune system function. This is important from an aging point of view for two reasons. First, immunoprecipitations or immunoproducts can be damaging to nerve cells, and have been implicated as being responsible for age-related neurodegeneration in the brain in general, and in Alzheimer's disease specifically, but this is a new finding for the cochlea and age-related hearing loss – presbycusis. Second, autoimmune problems, where the immune system starts attacking its own nerve cells, is another leading candidate for a causative factor in neurodegenerative aging conditions. These immune products are likely to come from the vascular supply to the cochlea, yet may be a causative component for age-related hearing loss due to the resultant damage to the cochlea sensory cells.

There are 3 genes having to do with post-translational protein changes, including protein binding properties, with two of these genes involved in carbohydrate metabolism (sugar/glucose binding in mitochondria for cellular respiration). These genes are related to the production of reactive oxygen species (ROS), which damage nerve cells, and have been implicated in age-related neurodegenerative disorders, and in cases of cochlear sensorineural hearing loss. For example, problems in cellular respiration can lead to accumulation of toxic intracellular substances, causing damage to sensory cell structures and abnormal metabolic processing along with increased levels of ROS [31-33].

The last gene, involved in mammary gland functioning, showed a significant increase with age. A closer inspection of the expression levels for this gene have shown that the observed effect cannot be attributed to the presence of outliers in the data. Although not directly involved in sensory functioning, this gene may change its espression as part of general degenerative processes in inner ear. An error in this gene annotation cannot be ruled out as well. This observation is definitely worth another look.

The above-described initial observations are quite provocative, in that we have several groupings of genes that have important functional significance for aging and hearing, including important aspects of cochlear, inner ear functioning. These animal-model gene-array investigations are quite useful for guiding human genetics experiments aimed at identifying candidate genes involved in the susceptibility and progression of human age-related hearing loss and other age-dependent neurosensory disorders.

Regarding methodological aspects of this paper, we would like to note that a pertinent multivariate method for selection of differentially expressed genes should include two components: finding subsets of candidate genes that jointly separate the classes (states) under comparison and testing statistical significance of this separation; the latter does not necessarily refer to characteristics of a classification (allocation) rule such as classification error rates. We also would like to stress that the problem of significance testing in the multivariate formulation is not equivalent to the problem of statistical classification (supervised learning). While closely related, these problems are fundamentally different. For example, the use of the classification error rate as a criterion for selection of important variables is appropriate where the aim is to form a discriminant rule for the subsequent outright allocation of unclassified samples to one of the known classes. A very good separation between classes can sometimes be provided by looking at a single feature variable (gene) so that the classification error rate is difficult to reduce further by including other (probably quite significant) variables in the rule. However, one would like to keep the chance of missing other interesting variables to a minimum. The problem dealt with in this paper is not that of classification or prediction. Our method is designed to find gene combinations that change in concert (as a set) their expression due to some biological factors. The problem thus formulated reduces to that of significance testing.

It must be emphasized that our method is designed not only to identify sets of genes whose interrelationships differ but also those genes with marginal effects. More importantly, the method seeks to provide an alternative way of making a specific FWER-based multiple testing procedure less conservative and, to some extent, less dependent on the subset pivotality requirement (see [4] for definition), by extracting more information from the data. In addition, this approach can be used for ranking and clustering those genes that have been declared differentially expressed by univariate methods.

## Conclusions

A new algorithm for identifying differentially expressed gene combinations has been developed. This algorithm is built on the earlier proposed multivariate test statistic [6] and successive selection of differentially expressed sets of genes [5]. The algorithm includes an improved random search procedure designed to generate candidate gene combinations of a given size. Cross-validation is used to provide replication stability of the search procedure. A permutation two-sample test is used for significance testing. We design a multiple testing procedure to control the family-wise error rate when selecting significant combinations of genes that result from a successive selection procedure. A target set of genes is composed of all significant combinations selected via random search. The performance of the proposed search-and-testing procedure has been evaluated by computer simulations and analysis of replicated Affymetrix gene array data on age-related changes in gene expression in the inner ear of CBA mice.

## Methods

### Subjects

CBA mice from the University of Rochester vivarium served as subjects for this study who had similar environmental, non-ototoxic life histories. Subjects were mice of the following age groups: Young adult (N = 9, 3–4 months) and old (N = 12, 24–33 months). All animal procedures were approved the University of Rochester Committee on Animal Resources.

### Cochlear dissections

Subject groups of the present report had extensive behavioral and neurophysiological hearing testing prior to sacrifice, verifying that the old mice had age-related hearing loss. Mice were sacrificed by cervical dislocation. Then both cochleae for each mouse were immediately dissected using a Zeiss stereomicroscope. The cochleae were placed in cold saline for micro dissection of the cochlear partition (basilar membrane, organ of Corti and spiral ligament), and were then placed in cold Trizol. A detailed protocol for Trizol can be found at . All samples were stored at -80°C for microarray gene expression processing.

### Gene expression microarrays

The RNA quality was assessed by electrophoresis using the Agilent Bioanalyzer 2100. Between 200 ng and 2 ug of total RNA from each sample was used to generate a high fidelity cDNA, which was modified at the 3' end to contain an initiation site for T7 RNA polymerase, while 1 ug of cDNA was used in an in vitro transcription (IVT). 20 ug of full-length cRNA, from each mouse (age groups as described above), was fragmented. After fragmentation, the cDNA, full-length cRNA, and fragmented cRNA were analyzed by electrophoresis using the Agilent Bioanalyzer 2100 to assess the appropriate size distribution prior to microarray hybridization. Detailed protocols for sample preparation using the Ambion MessageAmp protocol can be found at . Affymetrix M430A High density oligonucleotide array set (A) which queried 20,000 murine probe sets was used. Each gene on the subarray is represented by 11 pairs of 25 mer oligonucleotides that span the coding region for the 20,000 genes and ESTs represented (clear overlapping of genes is evident). Each probe pair consists of a perfect match (PM) sequence that is complementary to the cDNA target, and a miss-match (MM) sequence that has a single base pair mutation in a region critical for target hybridization; this sequence serves as a control for non-specific hybridization. Staining and washing of all arrays was performed in the Affymetrix fluidics module per manufacturer's protocol. Streptavidin phycroerythrin stain (SAPE, Molecular Probes) was the fluorescent conjugate used to detect hybridized target sequences. All arrays in this study were assessed for "array performance" prior to data analysis.

### Methods for data analysis and computer simulations

The methodology of data analysis and design of computer simulations have been described at length in the preceding sections. The relevant software for data analysis and simulations is included in the Additional Material Files [see the folder "MultivariateSearch"]. Here we supplement this information with a description of the generator of multivariate exchangeable normal random vectors which we used in our simulations.

Suppose we want to generate a normal random vector **X **in **R**^*d *^with mean vector **M **∈ **R**^*d *^and covariance matrix Σ whose entries are *σ*^2 ^and *ρσ*^2 ^on and off diagonal, respectively. It is well-known that **X **can be represented in the form

**X **= **M **+ **CZ**,

where **Z **is the standard normal vector with mean **0 **in **R**^*d *^and **C **is a *d *× *d *matrix with **CC**^*T *^= Σ. (Here **C**^*T *^denotes the transpose of **C**.) The matrix **C **may be chosen symmetric and can be computed using well-known algebraic procedures. However, our matrix Σ has a special structure:

Σ = (1 - *ρ*)*σ*^2^**I**_*d *_+ *ρσ*^2^**1**_*d *× *d*_,

where **I**_*d *_is a unit matrix of size *d *and **1**_*d *× *d *_is a square matrix with all the *d*^2 ^entries being equal to 1. Using this we look for **C **of the same form:

**C **= *α***I**_*d *_+ *β***1**_*d *× *d*_.

From the relations **C**^2 ^= Σ and  we have *α*^2 ^= *σ*^2^(1 - *ρ*), 2*αβ *= *ρσ*^2^, so that



## Authors' contributions

YX is responsible for the computational component of this study. He also participated in the methodology development. LK, AG, and AY have equally contributed to various methodological aspects of the proposed multivariate analysis. RF provided experimental data and biological interpretation of the net results of data analysis.

## Supplementary Material

Additional File 1 The additional folder "MultivariateSearch" includes the following three sub-folders: 1. SAO _Simulation 2. SRS_Simulation 3. TSSearch Each subfolder contains a Unix executable file. The executable file "SASearch" implements the algorithm based on simulated annealing optimization. The executable file "SRSearch" implement the version based on simple random search. The exectuable file "TSSearch" for the two-stage search is is located in the sub-folder "TSSearch". Each sub-folder also contains two input files. The file "simulation04_UI.txt" is an input file for data analysis. Suppose the data file is named xxxx.marr, then the input file should be named as xxxx_UI.txt. To analyze the data from the file xxxx.marr, type: [Executable file] xxxx or [Executable file] 0 xxxx. The input file "simulation04_ui.txt" is designed for simulation experiments. To conduct simulations, one has to prepare an input file with the name: XXX_simu_ui.txt, where XXX is a string that follows the naming convention of computer files. An input file for data analysis with the name XXX_ui.txt is also needed. To run simulations, type: [executable file] 1 xxxx. Click here for file

## Figures and Tables

**Table 1:** Proportions of correct discoveries for each gene in the target set.

Gene	SRS: 8 starts, 2500 steps		SRS: 16 starts, 3600 steps	
	
	Correct discovery *μ *= 1, *σ *= 0.5	Correct discovery *μ *= 1, *σ *= 1	Correct discovery *μ *= 1, *σ *= 0.5	Correct discovery *μ *= 1, *σ *= 1
1	100%	100%	100%	100%
2	100%	100%	100%	100%
3	100%	100%	100%	100%
4	100%	100%	100%	100%
5	100%	97%	100%	99%
6	100%	99%	100%	100%
7	100%	100%	100%	100%
8	100%	100%	100%	100%
9	99%	78%	100%	76%
10	99%	76%	100%	78%
11	96%	72%	100%	74%
12	97%	74%	100%	72%

## References

[B1] Almudevar A (2003). A simulated annealing algorithm for maximum likelihood pedigree reconstruction. Theoretical Population Biology.

[B2] Dudoit S, Shaffer JP, Boldrick JC (2003). Multiple hypothesis testing in microarray experiments. Statistical Science.

[B3] Pesarin F (2001). Multivariate Permutation Tests: With Applications in Biostatistics.

[B4] Westfall PH, Young S (1993). Resampling-Based Multiple Testing.

[B5] Szabo A, Boucher K, Jones D, Klebanov L, Tsodikov A, Yakovlev A (2003). Multivariate exploratory tools for microarray data analysis. Biostatistics.

[B6] Szabo A, Boucher K, Carroll W, Klebanov L, Tsodikov A, Yakovlev A (2002). Variable selection and pattern recognition with gene expression data generated by the microarray technology. Mathematical Biosciences.

[B7] Zinger AA, Klebanov LB, Kakosyan AV (1999). Characterization of distributions by mean values of statistics in connection with some probability metrics. Stability Problems for Stochastic Models.

[B8] Chilingaryan A, Gevorgyan N, Vardanyan A, Jones D, Szabo A (2002). Multivarite approach for selecting sets of differentially expressed genes. Mathematical Biosciences.

[B9] Ambroise C, McLachlan GJ (2002). Selection bias in gene extraction on the basis of microarray gene-expression data. Proceedings of the National Academy of Sciences USA.

[B10] Klebanov L, Gordon A, Xiao Y, Land H, Yakovlev A (2004). A new test statistic for testing two-sample hypotheses in microarray data analysis. Technical Report.

[B11] Bolstad BM, Irizarry RA, Astrand M, Speed TP (2003). A comparison of normalization methods for high density oligonucleotide array data based on variance and bias. Bioinformatics.

[B12] Irizarry RA, Gautier L, Cope LM, Parmigiani G, Garrett ES, Irizarry RA, Zeger SL (2003). An R package for analyses of Affymetrix oligonucleotide arrays. The Analysis of Gene Expression Data.

[B13] Corso JF (1980). Age correction factor in noise-induced hearing loss: a quantitative model. Audiology.

[B14] Gates GA, Caspary DM, Clark W, Pillsbury HC, Brown SC, Dobie RA (1989). Presbycusis. Otolaryngol Head Neck Surg.

[B15] Gelfand SA, Piper N, Silman S (1985). Consonant recognition in quiet as a function of aging among normal hearing subjects. J Acoust Soc Am.

[B16] Gelfand SA, Piper N, Silman S (1986). Consonant recognition in quiet and in noise with aging among normal hearing listeners. J Acoust Soc Am.

[B17] Lonsbury-Martin BL, Cutler WM, Martin GK (1991). Evidence for the influence of aging on distortion product otoacoustic emissions in humans. J Acoust Soc Am.

[B18] Lonsbury-Martin BL, Martin GK, Probst R, Coats AC (1987). Acoustic distortion products in rabbit ear canal. I. Basic features and physiological vulnerability. Hear Res.

[B19] Probst R, Lonsbury-Martin BL, Martin GK (1991). A review of otoacoustic emissions. J Acoust Soc Am.

[B20] Willott JF (1986). Effects of aging, hearing loss, and anatomical location on thresholds of inferior colliculus neurons in C57BL/6 and CBA mice. J Neurophysiol.

[B21] Willott JF (1991). Aging and the auditory system: Anatomy, physiology, and psychophysics.

[B22] Frisina DR, Frisina RD (1997). Speech recognition in noise and presbycusis: relations to possible neural mechanisms. Hear Res.

[B23] Frisina DR, Frisina RD, Snell KB, Burkard R, Walton JP, Ison JR, Hof PR, Mobbs CV (2001). Auditory temporal processing during aging. Functional Neurobiology of Aging.

[B24] Frisina RD, Hof PR, Mobbs CV (2001). Anatomical and neurochemical bases of presbycusis. Functional Neurobiology of Aging.

[B25] Jacobson M, Kim SH, Romney J, Zhu X, Frisina RD (2003). Contralateral suppression of distortion-product otoacoustic emissions declines with age: A comparison of findings in CBA mice with human listeners. Laryngoscope.

[B26] Guimaraes P, Zhu X, Cannon T, Kim SH, Frisina RD (2004). Sex differences in distortion product otoacoustic emissions as a function of age in CBA mice. Hear Res.

[B27] Gates GA, Couropmitree NN, Myers RH (1999). Genetic associations in age-related hearing thresholds. Arch Otolaryngol Head Neck Surg.

[B28] Kelley PM, Harris DJ, Comer BC, Askew JW, Fowler T, Smith SD, Kimberling WJ (1998). Novel mutations in the connexin 26 gene (GJB2) that cause autosomal recessive (DFNB1) hearing loss. Am J Hum Genet.

[B29] Kelsell DP, Dunlop J, Stevens HP, Lench NJ, Liang JN, Parry G, Mueller RF, Leigh IM (1997). Connexin 26 mutations in hereditary non-syndromic sensorineural deafness. Nature.

[B30] Kikuchi T, Adams JC, Miyabe Y, So E, Kobayashi T (2000). Potassium ion recycling pathway via gap junction systems in the mammalian cochlea and its interruption in hereditary nonsyndromic deafness. Med Electron Microsc.

[B31] Manna SK, Zhang HJ, Yan T, Oberley LW, Aggarwal BB (1998). Over-expression of manganese superoxide dismutase suppresses tumor necrosis factor-induced apoptosis and activation of NF- and activated protein-1. J Biol Chem.

[B32] Frisina ST, Mapes F, Kim SH, Frisina DR, Frisina RD (2003). Comprehensive characterization of hearing loss in aged diabetics. Paper presented at Society for Neuroscience 33rd Annual Meeting New Orleans, LA.

[B33] Gries A, Herr A, Kirsch S, Gunther C, Weber S, Szabo G, Holzmann A, Bottiger BW, Martin E (2003). Inhaled nitric oxide inhibits platelet-leukocyte interactions in patients with acute respiratory distress syndrome. Crit Care Med.

